# Changes in Oral Health and Dental Esthetic in Smokers Switching to Combustion-Free Nicotine Alternatives: Protocol for a Multicenter and Prospective Randomized Controlled Trial

**DOI:** 10.2196/53222

**Published:** 2024-02-23

**Authors:** Gianluca Conte, Sebastiano Antonio Pacino, Salvatore Urso, Doris Greiling, Pasquale Caponnetto, Eugenio Pedullà, Luigi Generali, Ugo Consolo, Vittorio Checchi, Stefan Gospodaru, Gheorghe Bordeniuc, Valeriu Fala, Jan Kowalski, Maciej Nowak, Renata Górska, Amaliya Amaliya, Iain Chapple, Michael Milward, Robert Maclure, Gianna Maria Nardi, Riccardo Polosa

**Affiliations:** 1 Addendo srl, Dental Clinic Catania Italy; 2 ECLAT Srl, Spin-off of the University of Catania Catania Italy; 3 Department of Biological, Geological and Environmental Sciences University of Catania Catania Italy; 4 Forschungsdock CRO GmbH Schenefeld Germany; 5 Center of Excellence for the Acceleration of HArm Reduction University of Catania Catania Italy; 6 Department of Educational Sciences, Section of Psychology University of Catania Catania Italy; 7 Department of General Surgery and Medical-Surgical Specialties University of Catania Catania Italy; 8 Department of Surgery, Medicine, Dentistry and Morphological Sciences with Transplant Surgery, Oncology and Regenerative Medicine Relevance University of Modena and Reggio Emilia Modena Italy; 9 Faladental Chișinău Republic of Moldova; 10 Department of Periodontology Medical University of Warsaw Warsaw Poland; 11 Department of Periodontology, Faculty of Dentistry Universitas Padjadjaran West Java Indonesia; 12 Periodontal Research Group The University of Birmingham & Birmingham Community Healthcare Trust Birmingham United Kingdom; 13 R Maclure Clinical Research Ltd Irby, Wirral United Kingdom; 14 Department of Dental and Maxillofacial Sciences Sapienza University Rome Italy; 15 Department of Clinical and Experimental Medicine University of Catania Catania Italy

**Keywords:** electronic cigarettes, heated tobacco products, tobacco harm reduction, smoking, oral health, gingivitis, periodontitis, Modified Gingival Index, MGI, dental plaque imaging, dental shade, smartphone, mobile phone

## Abstract

**Background:**

Although the detrimental effects of conventional combustible cigarettes on oral health and dental esthetics are well known, there is limited information about the long-term impact of combustion-free nicotine alternatives (C-F NA) such as e-cigarettes or heated tobacco products.

**Objective:**

This multicenter, prospective, 3-parallel-arm randomized controlled trial will investigate whether switching from combustible cigarettes to C-F NA will lead to measurable improvements in oral health parameters and dental esthetics over 18 months in adult smokers with limited gum disease.

**Methods:**

Regular smokers not intending to quit and without clinical signs of periodontitis will be randomly assigned (1:4 ratio) to either standard of care with brief cessation advice (control group; arm A) or C-F NA use (intervention group; arm B). The study will also include a reference group of never smokers (reference group; arm C). The primary end point is the change in the Modified Gingival Index (MGI) score from baseline between the control arm (arm A) and the intervention arm (arm B) at the 18-month follow-up. In addition, the study will analyze the within- and between-group (arms A, B, and C) changes in MGI assessment, plaque imaging, dental shade quantitation, tooth stain scores, and oral health–related quality of life questionnaires measured at each study time point. All participants will attend a total of 7 clinic visits: screening, enrollment, and randomization (visit 0); baseline visit—day 14 (visit 1); day 90 (visit 2); day 180 (visit 3); day 360 (visit 4); and day 540 (visit 5). This multicenter study will be conducted in 4 dental clinics in 4 countries. The statistical analysis will involve descriptive statistics for continuous and categorical data. Primary end points will undergo tests for normality and, based on distribution, either a 2-sided *t* test or Mann-Whitney *U* test. Linear mixed model with random factors center and study arms by center will also be applied. Secondary end points, including MGI assessment and quality of life, will be subjected to similar tests and comparisons. Only if one value of the parameter MGI is missing after day 1, the last available observation will be carried forward. The analysis will be performed on the substituted data. Secondary parameters will not have missing value replacement.

**Results:**

Participant recruitment began in October 2021, and enrollment was completed in June 2023. Results will be reported in 2025.

**Conclusions:**

This will be the first study to provide key insights into oral health benefits or risks associated with using C-F NA in smokers who are seeking alternatives to cigarette smoking.

**Trial Registration:**

ClinicalTrials.gov NCT04649645; https://clinicaltrials.gov/ct2/show/NCT04649645

**International Registered Report Identifier (IRRID):**

DERR1-10.2196/53222

## Introduction

### Background

Periodontal diseases, commonly including gingivitis and periodontitis, arise in response to the accumulation of oral bacteria in the gingival sulcus and are characterized by tissue changes in the periodontium that occur as part of the inflammatory process [1]. Gingivitis is characterized by inflammation such as redness, swelling, or bleeding on gentle provocation of the gingival sulcus, whereas periodontitis exhibits increased probing depth, clinical attachment loss, and radiographic alveolar bone loss, reflecting the destructive aspects of the disease process [2,3].

Periodontal disease is a well-known independent risk factor for cardiovascular disease [4,5], and reducing gingivitis and periodontitis is likely to have an overall positive impact on human health in general.

There are multiple risk factors for periodontal disease, and cigarette smoking is considered among the key independent risk factors for the development and progression of periodontal disease [6-9]. Depending on the disease definition and extent of exposure to cigarette smoke, the risk of developing destructive periodontal disease is 5- to 20-fold higher in smokers than in nonsmokers [10]. Cigarette smokers appear to be prone to more severe periodontal manifestations even after adjusting for age, education level, history of diabetes, BMI, alcohol consumption, perceived mental stress, and oral hygiene levels [11]. In addition to periodontal diseases, cigarette smoking can cause visible dental manifestations, including dental discoloration and tobacco stains, the intensity of which mainly depends on smoking duration and frequency [12]. In a large cross-sectional study in the United Kingdom [13], smokers were more likely to report dental discoloration and being dissatisfied with their own tooth color compared with nonsmokers. Dissatisfaction with teeth appearance (because of enamel discoloration and tobacco stains) is often perceived as a significant social problem for smokers [14,15].

The importance of dental care and oral health for healthy longevity is emphasized in the 2022 declaration of the World Health Organization (WHO) for which greater advocacy is needed to increase the prominence of oral health on the global health agenda. Moreover, reducing oral health issues calls for stronger policies addressing the determinants of oral diseases and no communicable diseases and to tackle inequalities through inclusive universal health care access [16]. More recently, the WHO has provided policy recommendations for integrating brief tobacco interventions into oral health programs in primary care in accordance with the WHO Oral Health Program and as part of the WHO Global action plan on the prevention and control of noncommunicable diseases, particularly pertaining to the needs of low- and middle-income countries [17].

Although it is clear that abstaining from smoking will have beneficial effects on oral health and overall dental esthetic, most smokers are reluctant to seek formal treatment to stop smoking, with the vast majority making attempts to quit without assistance [18,19]. Consequently, novel and efficient approaches are required.

Although not authorized as medications for smoking cessation, combustion-free technologies for nicotine delivery, such as e-cigarettes (ECs) and heated tobacco products (HTPs), have become de facto harm reduction tools from cigarette smoke [20,21] and aid in quitting smoking [22,23].

Combustion-free technologies for nicotine delivery such as ECs and HTPs offer substantial reduction in exposure and harm to harmful constituents compared with tobacco cigarettes [24-27], but there is limited information about the long-term impact on oral health and dental esthetics in people who use combustion-free nicotine alternatives (C-F NA).

### Objectives

This multicenter, prospective, randomized, controlled, 3–parallel arm trial will be the first to determine whether adult smokers who switch to C-F NA will experience measurable improvements in oral health parameters and dental esthetics. A group of never smokers will also be recruited for comparative measures of the study parameters.

Data from this study will provide valuable insights into the overall potential of C-F NA in reducing the risk of periodontal diseases and consequential cardiovascular risk. In addition, the findings may have important implications for reducing the smoking burden globally, especially for smokers for whom bad breath or poor dental esthetic is a significant concern. For these individuals, an oral-centric narrative (such as achieving a healthier and brighter smile) may serve as a more compelling reason to quit smoking than the fear of future lung cancer or cardiopulmonary diseases.

## Methods

This is a multicenter, 3-parallel-arm, randomized controlled trial of 18 months duration designed to assess whether cigarette smokers switching to C-F NA will undergo measurable improvements in oral health parameters and dental esthetics as a consequence of avoiding exposure to cigarette smoke. This study aims to assess differences in study end points of oral health parameters and dental esthetics at multiple study time points between smokers switching to C-F NA and smokers who continue to smoke.

### Study Population

Eligible participants will be healthy adult regular cigarette smokers (self-reported daily smoking of >10 cigarettes per day for at least 5 consecutive years) and never smokers without clinical signs of periodontitis.

Smokers will be offered access to free smoking cessation programs. Only those who refuse to participate in the smoking cessation program and express willingness to switch to C-F NA will be eligible for randomization.

Never smokers (those who have smoked <100 tobacco cigarettes in their lifetime) will also be included as a reference group.

Participants will be required to satisfy all the inclusion criteria as presented in Textbox 1.

**Inclusion and exclusion criteria.**

**Inclusion criteria for smokers (study arms A and B)**

- Adults (aged 18-50 years)
- Cigarette smokers of ≥10 cigarettes per day
- Smoked for at least 5 consecutive years before screening
- Verified smoking status by exhaled breath carbon monoxide (CO) ≥7 ppm at screening
- Willingness to switch to a combustion-free nicotine alternatives, if required by the randomization
- Refusal to participate in smoking cessation programs
- Presence of at least 10 natural anterior teeth in total (cuspid to cuspid and lower and upper jaw)
- Healthy participants, not taking regular medications for chronic medical conditions
- Accepting to comply with the requirements of the study, including installing an app on their smartphone

**Inclusion criteria for never smokers (study arm C)**

- Adults (aged 18-50 years)
- Have not smoked >100 tobacco cigarettes in their lifetime
- Verified nonsmoking status by exhaled breath CO <7 ppm at screening
- Presence of at least 10 natural anterior teeth in total (cuspid to cuspid and lower and upper jaw)
- Healthy participants, not taking regular medications for chronic medical conditions
- Accepting to comply with the requirements of the study, including installing an app on their smartphone

**Exclusion criteria for smokers (study arms A and B) and never smokers (study arm C)**

- Significant oral soft tissue pathology or any type of gingival overgrowth other than plaque-induced gingivitis
- Periodontitis based on the 2017 World Workshop on the Classification of Periodontal and Peri-Implant Diseases and Conditions [28], which requires the following:
  Detectable interdental clinical attachment loss ≥3 mm at ≥2 nonadjacent teeth
  Buccal or oral clinical attachment loss ≥3 mm with pocketing ≥3 mm detectable at ≥2 teeth
  Only participants with mild to moderate gingivitis will be recruited
- Fixed and removable orthodontic appliances or removable dentures
- Any other medical condition that, in the opinion of the principal investigator, would jeopardize the participant’s safety or diminish the validity of the study results
- A course of treatment with any medications that
  interfere with the cyclooxygenase pathway (eg, anti-inflammatory drugs including aspirin and ibuprofen) within 3 days before each visit
  are known to have antibacterial activity (eg, antibiotics) within 7 days before each visit
- Significant history of alcohol or drug abuse within 24 months before screening, as determined by the investigator
- For smokers, planning to quit smoking within the next 6 months
- For smokers, regular use of nicotine (eg, e-cigarettes, nicotine replacement therapy, nicotine pouches) or tobacco products (eg, heated tobacco products, oral smokeless) other than their cigarettes within 14 days of screening
- Pregnant or breastfeeding or intention to become pregnant during the study
- Active participation in another clinical trial

### Study Design

**Figure 1:**
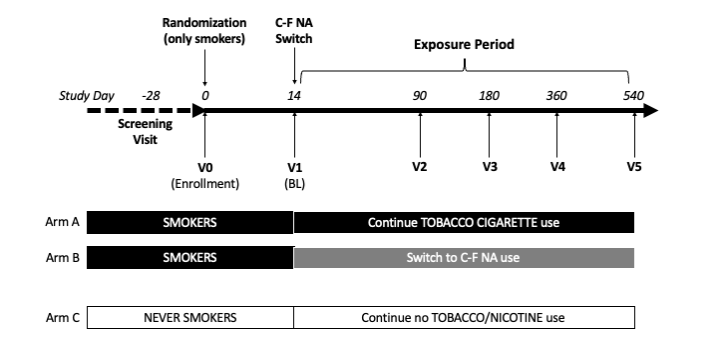
Study design. Smokers not intending to quit and without clinical signs of periodontitis were randomized to either standard care (arm A) or combustion-free nicotine alternatives (C-F NA; arm B). A reference group of never smokers will also be included but not randomized (arm C). Participants were prospectively reviewed at 4 dental clinics for cigarette consumption and C-F NA use, oral health and dental esthetic measurements, vital signs, and adverse events for up to 18 months.

This is an 18-month, prospective, multicenter, open-label study with 3 parallel arms, using randomization and control to assess a range of oral health and dental esthetic metrics (Figure 1). The primary objective is to compare these metrics between cigarette smokers transitioning to C-F NA and those who continue smoking.

Eligible smokers will be allocated randomly to either the control group (arm A), which will receive standard care inclusive of cessation counseling (ie, very brief advice [VBA]) or the intervention group (arm B), which will be given a C-F NA of their choice and will also receive VBA. Individuals who have never smoked will form a reference group (arm C). Throughout the study, participants from all 3 groups will undergo regular assessments of their oral health and dental esthetic. The study will be conducted in 4 dental clinics across 4 different countries (Italy, Poland, Moldova, and Indonesia).

The study protocol adheres to the guidelines stipulated by the Standard Protocol Items: Recommendations for Interventional Trials (SPIRIT) guidelines.

**Figure 2:**
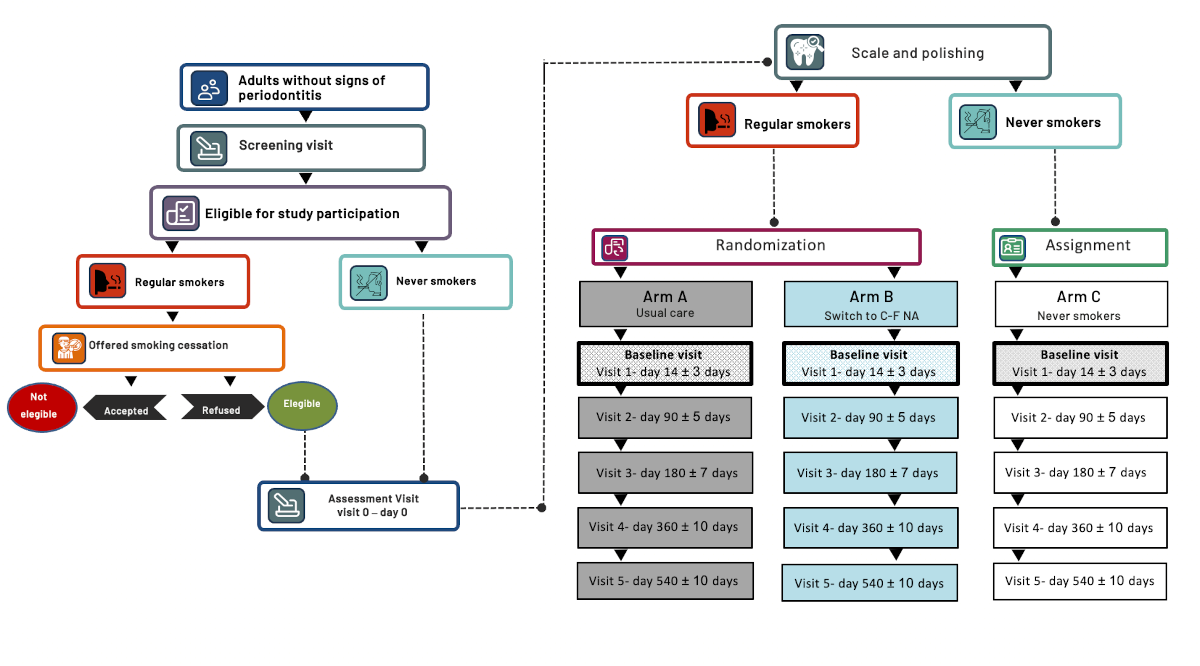
Study flow diagram of study participants. C-F NA: combustion-free nicotine alternatives.

The study flow is illustrated in Figure 2.

All participants will attend a total of 7 clinic visits: day −28 to day −1—screening; day 0—enrollment and randomization (visit 0); day 14—baseline visit (visit 1); day 90—week 12 (visit 2); day 180—week 24 (visit 3); day 360—week 52 (visit 4); day 540—week 76 (visit 5).

Screening visits will be performed within 28 days before enrollment to verify the eligibility criteria (visit 0; Figure 2). During the screening visit, sociodemographic data, medical and oral health history, detailed information about nicotine and tobacco consumption, and interest in trying C-F NA will be recorded. Screening for periodontal disease will be performed to exclude periodontitis, as per the definition of the guidelines of the 2017 Classification of Periodontal and Peri-implant Diseases and Conditions [28]. Smokers will be offered a smoking cessation program as per local guidelines. Any smokers who express the intention of booking for the cessation program or to quit smoking in the next 6 months will be urged to do so and will not be recruited in the study. Participants taking part in the study will be informed that they are free to quit smoking and withdraw from the study at any time. Moreover, smokers will be encouraged to quit smoking at every contact throughout the study.

Within 28 days of the screening visit, eligible participants will be invited to attend an enrollment visit (visit 0). Inclusion and exclusion criteria will be verified again. Smokers will be reminded of the risks associated with smoking before enrollment in the study and that they are free to voluntarily quit smoking and nicotine or withdraw from the study at any time. All participants will undergo a series of measurements (Tables 1-3) followed by dental scaling and polishing procedures to remove calculus, plaque, and stain.

Smokers not intending to quit will then be randomly assigned to either the control (arm A) or the intervention (arm B) group. The randomization sequence will be generated by a computer, with an allocation ratio of 1:4 (arm A: arm B) to accommodate for the estimated 25% proportion of participants achieving a sustained reduction in cigarette consumption of at least 90% in arm B (as detailed in the Statistical Considerations section). Smokers in the control group (arm A) will receive standard care, inclusive of cessation counseling (ie, VBA) at each study visit. Smokers in the intervention group (arm B) will have the option to try and choose a preferred C-F NA (ECs and HTPs) from a given pool of popular options present in the respective markets and will also receive VBA.

Individuals who have never smoked will be allocated to the reference group (arm C). Never smokers in the reference group will serve as comparators for assessing any background changes in the primary study end point (ie, Modified Gingival Index [MGI]) that may occur over time. Although never smokers will not undergo randomization, efforts will be made to achieve a comparable distribution of sex and age across all study arms.

The trial is designed as an unblinded trial because of the nature of the intervention, where participants and trial staff cannot be blinded to the specific intervention being provided. However, data analyses will be conducted blind to study arms allocation. All other trial staff who have access to outcome data will remain blinded until prespecified data analyses will be completed.

Baseline measurements will be performed at visit 1 (baseline visit) and 14 days after scaling and polishing procedures at enrollment (visit 0). Study measurements will include MGI assessment, plaque imaging, dental shade quantitation, tooth stain scores, and oral health–related quality of life questionnaires (Tables 1-3).

Smokers in arm B will be provided with a C-F NA of their choice. They will receive training and counseling on how to use the chosen C-F NA and will be provided with a full 2-week supply of the required consumables (cartridges or pods or e-liquid refill bottles for ECs and tobacco sticks for HTPs). Participants wishing to use an HTP will receive one kit and a supply of tobacco sticks of their choice. They will receive the number of tobacco sticks per day corresponding to the number of cigarettes smoked per day at baseline. Participants wishing to use a vaping product will receive one vaping kit and a supply of e-liquids of their choice. Products will be supplied at each subsequent visit throughout the study according to the study product supply schedule in Table 4.

After the baseline visit, participants will be invited to attend 4 more clinical visits (visits 2-5) to repeat study measurements (Tables 1-3).

Throughout the study, monitoring of cigarette consumption, daily C-F NA use, and oral hygiene routine will be carried out regularly using the tracker app and personal diaries. Monitoring of cigarette consumption and daily C-F NA use will also be achieved by asking participants in arm B to return all empty, partly used, and unused consumables (tobacco sticks, EC cartridges, and e-liquid refill bottles) at each study visit. The tracker app will also identify any protocol violations, collect adverse events (AEs), and send reminders (eg, next scheduled appointment, study restrictions, and instructions) throughout the study duration. The use of a dedicated tracker app adds an innovative element to continuously collect data and enhances adherence to the study protocol.

Before each study visit (visits 1-5), all participants will be required to refrain from scaling and polishing procedures, avoid modifying their habitual oral hygiene (eg, mouthwash, mouth rinse, and interdental floss), refrain from flossing for at least 72 hours before each study visit, refrain from mouth rinsing for at least 24 hours before each study visit, refrain from tooth brushing for at least 2 hours before each study visit, refrain from eating and drinking (except water) for at least 2 hours before each visit, and abstain from smoking for 2 hours before each visit. This is to minimize any potential interference with study outcomes and to maintain consistency in data collection. Throughout the study, any modifications in the oral hygiene routine of participants will be recorded using the tracker app.

A detailed description of the measurements that will be specifically performed at each trial visit is provided in Multimedia Appendix 1.

**Table 1:** Study schedule of procedures (control group—arm A).

Procedure	Screening	Enrollment (V0); day 0	Baseline (V1); day 14±3	Week 12 (V2); day 90±5	Week 24 (V3), day 180±7	Week 52 (V4); day 360±7	Week 76 (V5); day 540±7
Eligibility criteria check	✓	✓					
Medical and oral health Hx^a^	✓						
Smoking Hx	✓						
Intention to quit smoking	✓						
Smoking cessation advice	✓	✓	✓	✓	✓	✓	✓
Cigarette consumption	✓	✓	✓	✓	✓	✓	✓
Exhaled CO^b^	✓	✓	✓	✓	✓	✓	✓
Interest to switch to C-F NA^c^	✓						
Informed consent		✓					
FTND^d^		✓					
Oral hygiene check	✓	✓	✓	✓	✓	✓	✓
Periodontal examination	✓						
Scaling and polishing		✓					✓
Randomization		✓					
Tracker app installation		✓					
BP^e^, HR^f^, weight, height, and BMI		✓	✓	✓	✓	✓	✓
MGI^g^ assessment		✓	✓	✓	✓	✓	✓
Plaque score imaging		✓	✓	✓	✓	✓	✓
Dental shade quantitation		✓	✓	✓	✓	✓	✓
Tooth stain assessment		✓	✓	✓	✓	✓	✓
QoL^h^ questionnaires (OHQoL^i^ and EQ VAS^j^)		✓	✓	✓	✓	✓	✓
Safety reporting		✓	✓	✓	✓	✓	✓

^a^Hx: history.

^b^CO: carbon monoxide.

^c^C-F NA: combustion-free nicotine alternatives.

^d^FTND: Fagerström Test for Nicotine Dependence.

^e^BP: blood pressure.

^f^HR: heart rate.

^g^MGI: Modified Gingival Index.

^h^QoL: Quality of Life.

^i^OHQoL: Oral Health Quality of Life.

^j^EQ VAS: EuroQoL Visual Analog Scale.

**Table 2:** Study schedule of procedures (intervention group—arm B).

Procedure	Screening	Enrollment (V0); day 0	Baseline (V1); day 14±3	Week 12 (V2); day 90±5	Week 24 (V3); day 180±7	Week 52 (V4); day 360±7	Week 76 (V5); day 540±7
Eligibility criteria check	✓	✓					
Medical and oral health Hx^a^	✓						
Smoking Hx	✓						
Intention to quit smoking	✓						
Smoking cessation advice	✓	✓	✓	✓	✓	✓	✓
Cigarette consumption	✓	✓	✓	✓	✓	✓	✓
Exhaled CO^b^	✓	✓	✓	✓	✓	✓	✓
Interest to switch to C-F NA^c^	✓						
Informed consent		✓					
FTND^d^		✓					
Oral hygiene check	✓	✓	✓	✓	✓	✓	✓
Periodontal examination	✓						
Scaling and polishing		✓					✓
Randomization		✓					
Familiarization with C-F NA			✓				
C-F NA use				✓	✓	✓	✓
Tracker app installation		✓					
BP^e^, HR^f^, weight, height, and BMI		✓	✓	✓	✓	✓	✓
MGI^g^ assessment		✓	✓	✓	✓	✓	✓
Plaque score imaging		✓	✓	✓	✓	✓	✓
Dental shade quantitation		✓	✓	✓	✓	✓	✓
Tooth stain assessment		✓	✓	✓	✓	✓	✓
QoL^h^ questionnaires (OHQoL^i^ and EQ VAS^j^)		✓	✓	✓	✓	✓	✓
Safety reporting		✓	✓	✓	✓	✓	✓

^a^Hx: history.

^b^CO: carbon monoxide.

^c^C-F NA: combustion-free nicotine alternatives.

^d^FTND: Fagerström Test for Nicotine Dependence.

^e^BP: blood pressure.

^f^HR: heart rate.

^g^MGI: Modified Gingival Index.

^h^QoL: Quality of Life.

^i^OHQoL: Oral Health Quality of Life.

^j^EQ VAS: EuroQoL Visual Analog Scale.

**Table 3:** Study schedule of procedures (reference group—arm C).

Procedure	Screening	Enrollment (V0); day 0	Baseline (V1); day 14±3	Week 12 (V2); day 90±5	Week 24 (V3); day 180±7	Week 52 (V4); day 360±7	Week 76 (V5); day 540±7
Eligibility criteria check	✓	✓					
Medical and oral health Hx^a^	✓						
Smoking Hx	✓						
Cigarette consumption	✓	✓	✓	✓	✓	✓	✓
Exhaled CO^b^	✓						
Informed consent		✓					
Oral hygiene check	✓	✓	✓	✓	✓	✓	✓
Periodontal examination	✓						
Scaling and polishing		✓					✓
Assignment to arm C		✓					
Tracker app installation		✓					
BP^c^, HR^d^, weight, height, and BMI		✓	✓	✓	✓	✓	✓
MGI^e^ assessment		✓	✓	✓	✓	✓	✓
Plaque score imaging		✓	✓	✓	✓	✓	✓
Dental shade quantitation		✓	✓	✓	✓	✓	✓
Tooth stain assessment		✓	✓	✓	✓	✓	✓
QoL^f^ questionnaires (OHQoL^g^ and EQ VAS^h^)		✓	✓	✓	✓	✓	✓
Safety reporting		✓	✓	✓	✓	✓	✓

^a^Hx: history.

^b^CO: carbon monoxide.

^c^BP: blood pressure.

^d^HR: heart rate.

^e^MGI: Modified Gingival Index.

^f^QoL: Quality of Life.

^g^OHQoL: Oral Health Quality of Life.

^h^EQ VAS: EuroQoL Visual Analog Scale.

**Table 4:** Study schedule of combustion-free nicotine alternatives (C-F NA) supply and use checks (only for arm B).

Procedure	Screening	Enrollment (V0)	Baseline (V1)	Week											
				4	8	12 (V2)	20	24 (V3)	32	40	48	52 (V4)	60	68	76 (V5)
C-F NA use			✓	✓	✓	✓	✓	✓	✓	✓	✓	✓	✓	✓	✓
Provide C-F NA device			✓												
Hand out 2 weeks supply of consumables^b^			✓												
Hand out 4 weeks supply of consumables^a^				✓	✓		✓				✓				
Hand out 2×4 weeks supply of consumables^b^						✓		✓	✓	✓		✓	✓	✓	✓
Product use checks (collect used and unused consumables^b^)				✓	✓	✓	✓	✓	✓	✓	✓	✓	✓	✓	✓

^a^Participants wishing to use a heated tobacco product will receive one kit and supply of tobacco sticks of their choice; they will receive the number of tobacco sticks per day corresponding to the number of cigarettes smoked per day at baseline. Participants wishing to use a vaping product will receive one vaping kit and supply of e-liquids of their choice.

### Study End Points

#### Primary End Points

The primary objective of the study is to compare changes in MGI by Lobene et al [29] between baseline and end of the study (ie, 18 months’ time point) among participants who continue to smoke tobacco cigarettes (arm A) and participants who switch to C-F NA (arm B).

#### Secondary End Points

In addition, the study will analyze the number of secondary objectives, including within- and between-group variations at each study time point from baseline for MGI assessment, plaque imaging, dental shade quantitation, tooth stain scores, and oral health–related quality of life questionnaires.

### Study Measurements and Calibration

A detailed description of the study measurements that will be specifically carried out at the dental clinic is provided in Multimedia Appendix 2.

All assessors will be trained on MGI scoring, tooth stain assessment, and the correct use of technologies and software used to quantify dental plaque, tooth discoloration, exhaled carbon monoxide monitoring, and electronic case report form (eCRF) use. Assessors will be periodically calibrated on MGI scoring and tooth stain assessment. To ensure consistency in the measurements, the same assessor will collect all study end points in the same participant over the entire study period.

### Data Monitoring and Study Safety

A trial monitoring plan will be developed based on the trial risk assessment and includes on-site monitoring. The clinical research organization will arrange an independent monitor, separate from the investigators and the sponsor, to ensure compliance with trial protocols and policies, participant protection, and accurate data collection.

AEs and serious AEs (SAEs; eg, those related to oral conditions, tobacco cigarette smoking, C-F NA use, nicotine withdrawal symptoms, or nicotine overdosing) will be recorded on the AE page of the eCRF throughout the study. Participants will be interviewed at each visit to investigate signs or symptoms. Signs or symptoms will be elicited at each visit by open questioning, such as “How have you been feeling since your last visit?” “How have you been feeling regarding your oral cavity?” Participants will also be encouraged to spontaneously report AEs occurring at any other time during the study via the dedicated mobile app or the communication channels provided for the study. The investigator must gather sufficient information to determine the outcome and causality of AEs and SAEs and promptly notify the competent authority, if necessary.

### Study Withdrawal

Participants may be withdrawn from the study prematurely for the following reasons: (1) experience an SAE, (2) develop a concurrent disease which at the discretion of the investigator no longer justifies the participant’s participation in this study, (3) sustain any uncorrectable protocol deviations during the study, (4) decide to stop their participation, (5) become pregnant, and (6) exhibit uncooperative behavior or nonattendance. After consultation with the clinical research organization, the investigator will notify participants’ discontinuation from the study.

### Data Collection and Protection

For data collection, each participant will be allocated an eCRF. Anonymized data from each study visit will be entered directly into the eCRF, which will then serve as a source document for the trial. This data entry process ensures that data are collected accurately and consistently at all study sites.

Each participant will be assigned a unique study identification number (participant ID). Participant’s personal data will not be linked to the research results, and only a limited number of members of the research team will have access to the decoding list that links participant IDs to their personal information. All information obtained during the study procedures, including participant data and personal details, will be treated as private and confidential in accordance with ethical and privacy regulations.

The trial will reach its formal conclusion on the date of the final visit of the last participant in the last country involved in the study.

### Ethical Considerations

The study will be conducted in accordance with the Principles of Good Clinical Practice and the Declaration of Helsinki. All 4 local ethics review boards reviewed and approved the study and, where appropriate, translated the relevant documentation (eg, informed consent form, participant’s information sheet). The trial is registered with ClinicalTrials.gov (NCT04649645). The study was approved by the ethics review board of the coordinating center, the Azienda Ospedaliera di Rilievo Nazionale e di Alta Specializzazione “Garibaldi” (697/CE; dated November 11, 2020). Informed consent was obtained from all the participants.

The intention is to disseminate the study results through articles in peer-reviewed journals and conference presentations. A summary of the results will be available on the study website for public access. The anonymized data will be available to researchers upon reasonable request.

### Statistical Considerations

#### Sample Size Calculation

For a valid sample size calculation, literature research was performed for the primary end point of the study (ie, MGI). Unfortunately, no data were available for such a study. However, data from studies with mouth rinse were available, and based on these studies [30,31], a mean MGI change of 0.08 with an SD of 0.18 of the MGI seemed relevant.

However, in these studies, higher mean MGI changes were observed because active treatment was administered, which was not the case in this study. Thus, several scenarios with different mean MGI changes and SDs will be applied.

The following hypotheses will be tested with a 1-sided independent *t* test:

H0: µA≤µB versus H1: µA>µB

where µA denotes the mean change MGI from baseline to after 18 months of regular smoking and µB denotes the mean MGI from baseline to after 18 months of C-F NA use.

For sample size calculation, SAS software (version 9.4; SAS Institute) was used and several scenarios were applied, assuming that the data are normally distributed. The results are summarized in Table 5, which presents the calculated sample sizes for the comparison of µA versus µB by a 1-sided independent *t* test, a significance level of 5%, and a power of 90%, depending on the expected MGI change and SD.

As no active treatment is given in this study, a lower effect than the observed values needs to be assumed. Therefore, a mean MGI change of 0.1 with an SD of 0.21 was chosen. Hence, a sample size of 77 patients per treatment group is required. Considering a dropout rate of 20% over 2 years’ observation phase [32,33], a sample size of at least 92 per treatment group is required. In addition, it is estimated that approximately 25% of those switching to C-F NA use will abstain or substantially (≥90%) reduce smoking [34], and only these participants deliver valid results. Therefore, 4 times more participants should be recruited in the C-F NA group (4 × 92 = 368). This results in a total sample size of 460 (92 in arm A+368 in arm B).

In arm C, we have determined a sample size of 40, empirically with no formal statistical analysis, as this cohort’s primary study end point (ie, MGI) is expected to be stable throughout the study.

**Table 5:** Summary table including the sample size for each arm of the study depending on the mean Modified Gingival Index (MGI) change and SD.

Nominal power	Mean MGI change	SD^a^				
		0.20	0.21	0.22	0.23	0.24
0.9	0.10	70	77	84	92	100
0.9	0.15	32	35	38	41	45
0.9	0.20	18	20	22	24	26

^a^Expected sample size is the same per each group and it is indicated by the rows (ie, 70-100, 32-45, and 18-26) according to the SD and MGI combinations.

#### Statistical Analyses

Statistical methods used in the analysis of this study will include descriptive statistics with the descriptive presentation of the total sample value, mean, SD, median, minimum, maximum, and 95% CIs for (pseudo) continuous data and categorical data. Further evaluations will be summarized in tables by counts and percentage of scores.

Statistical methods for primary study end points will include the following: the Shapiro-Wilk test of normality on calculated values (in case of normal distribution, a 2-sided independent *t* test and in case of nonnormal distribution: a 2-sided Mann-Whitney *U* test) and a linear mixed model with the random factors “center” and “study arms by center.”

Designated as study arm C, the never-smoker group will establish a baseline reference for the oral health parameters. This group is anticipated to provide insights into the underlying changes in the primary study end point (MGI) that may emerge over time in individuals who have never smoked.

Statistical methods for secondary study end points will include the following: for comparisons of each study arm for parameters such as MGI assessment, plaque imaging, dental shade quantitation, tooth stains scores, and oral health–related quality of life questionnaires, the Shapiro-Wilk test of normality on calculated values (in case of normal distribution, a 2-sided independent *t* test and in case of nonnormal distribution: a 2-sided Mann-Whitney *U* test); for comparisons of each assessment time for parameters such as MGI assessment, plaque imaging, dental shade quantitation, tooth stains scores, and oral health–related quality of life questionnaires, the Shapiro-Wilk test of normality on calculated values (in case of normal distribution, a 2-sided independent *t* test and in case of nonnormal distribution: a 2-sided Mann-Whitney *U* test); accounting for missing, unused, and spurious data.

If only one value of the parameter MGI is missing after day 1, the last available observation will be carried forward. The analysis will be performed on the substituted data. For secondary parameters, no replacement of missing values will be applied.

A generalized linear regression model will be used to adjust for all identified confounders. To identify possible predictors of primary and secondary study end points, a regression model will be estimated in which each study end point will be entered as the dependent variable. Possible predictors will be entered into the model as independent variables (including study group; age; gender; cigarette consumption; combustion-free nicotine delivery systems use; and frequency of personal oral hygiene, eg, tooth brushing, mouth washing, and dental flossing frequency) to assess the interactions. Following the results obtained from each regression model, comparisons between and within study groups will be performed using analysis of covariance adjusted for age, gender, and frequency of personal oral hygiene followed by the Tukey post hoc comparison test.

The goal of our study is to help smokers abstain from cigarette smoking to assess changes in oral health regardless of the product used. This study is not designed to compare the efficacy between HTPs and ECs in terms of smoking cessation. Nonetheless, we will consider a secondary analysis that takes into account the potential different impact of HTPs versus ECs in terms of oral health outcomes.

## Results

Participant recruitment began in October 2021, and enrollment has been completed in June 2023. Results will be reported in 2025.

## Discussion

### Overview

Although the negative effects of cigarette smoking on oral health and tooth discoloration are well known [35,36], there are only limited data about the impact of C-F NA such as ECs and HTPs. In particular, there are no long-term studies assessing the impact on oral health and teeth appearance when substituting conventional cigarettes for these combustion-free alternatives. This study was specifically designed to address these research questions. In particular, this study tests the hypothesis that avoiding exposure to cigarette smoke toxicants may translate into measurable amelioration in gingival response, dental plaque build-up, enamel discoloration, and tooth staining in participants with mild to moderate gingivitis by comparing participants who smoke tobacco cigarette with those who switch to using C-F NA or participants who never smoked.

In addition to its obvious relevance to health and esthetic concerns, this study seeks to expand on regulatory science. Regulatory authorities (eg, the US Food and Drug Administration) recommend investigating the oral health effects of novel tobacco and nicotine products to better understand their impact at individual and population levels as well as exploring additional study end points for the assessment of their short- and long-term effects in oral health studies [37]. Our study is designed to provide improved design knowledge for oral health switching studies and, most importantly, to streamline new innovative cutting-edge technologies that can be used for future studies on existing and emerging tobacco and nicotine products (including oral smokeless products).

The study design incorporates several significant and groundbreaking characteristics. First, we aim to enhance adherence to C-F NA and optimize overall compliance with the study’s instructions through the provision of a diverse range of products, encompassing the most popular options available in the market. This approach allows participants to tailor their own gratifying “nicotine experience” by selecting the C-F NA that aligns with their preferences. By doing so, we anticipate not only fostering a transition to the latest technology but also facilitating the reduction of cigarette smoking and bolstering long-term prevention of relapse [38-42]. It is worth highlighting that this aspect of personal choice is notably absent in studies of harm reduction. Furthermore, it is important to emphasize that the outcomes of this research will not be product specific, thereby minimizing constraints on generalizability.

Second, the study is being run in many different locations across the globe and so the challenge was to find a primary end point that could be measured by different operators in different sites, using a standardized measurement system that has been used previously and so has provenance. The index to be used had to be simple, reproducible, and relatively easy to compare between different sites and different operators. Ideally, the assessors should be able to calibrate the index to be used and compare the calibration results. The MGI is a widely used industry-standard index to determine changes in the gingival health of volunteers in clinical studies [29,43,44]. This index is simple, noninvasive, and reproducible, and it is hoped that it will be possible to train different examiners in different locations to align their assessments, thus making the results comparable. As the MGI is noninvasive, there is no gingival probing, and it is thought to be easier to calibrate examiners and compare kappa scores. Although subjective in nature, the lack of probing removes one of the variables present in using pressure to probe the gingivae. It is also thought to afford greater sensitivity in determining therapeutic efficacy [45]. This study will be the first to investigate the long-term impact of smoking or C-F NA intervention on MGI.

Third, the selection of secondary study end points is strategic because it considers what drives smokers to turn to cleaner nicotine and tobacco products. This is particularly persuasive for young adults, for whom a cardiovascular-cancer-respiratory risk–based narrative is either ineffective or even counterproductive, and for whom concern about bad breath and poor dental esthetic (because of enamel discoloration and “tar” stains) may be a much more significant reason to refrain from smoking. Although experimental work comparing the effects of C-F NA on tooth staining and discoloration has been published [46-48], this study is the first to consider this powerful narrative of oral health in a clinical trial; therefore, we have included innovative 21st century technologies for objective and consistent quantitation of dental shade discoloration (by calibrated spectrophotometry) and dental plaque changes (by digital imaging technology; quantitative light-induced fluorescence) among the study end points to investigate whether switching completely from cigarettes to C-F NA can improve gum health, reduce bad breath, and restore teeth appearance. Small-scale clinical trials have been recently conducted at the Center of Excellence for the Acceleration of Harm Reduction of the Catania University to confirm the validity and reproducibility of dental shade assessment by digital spectrophotometry and dental plaque quantitation by light-induced fluorescence technology in current, former, and never smokers [49,50].

Fourth, maximizing the magnitude of observable changes at the study end points is critically important. Given that several factors (including duration of smoking exposure, variations in oral hygiene practices, type of diet, and level of alcohol consumption) can significantly affect assessments of gum health and dental esthetics, we reduced baseline variability by using data obtained after the visit during which scaling and polishing was carried out. By removing dental plaque, calculus, and stains, we provide all study participants with the best possible oral health status (gum health and teeth appearance) at the beginning of the study. Considering that a 14-day interval is generally required to allow gingival restoration from tissue trauma caused by scaling and polishing [51], measurements that will be considered for this normalized baseline will be obtained 14 days after scaling and polishing. Any progressive change in gum health or tooth appearance will be compared with the reference data of this baseline. In addition to the inclusion of a full scale and polish at the beginning of the study in an attempt to maximize the magnitude of observable changes of study end points, it is also important to consider that the length of the study is adequate and that the study population will have to be one that allows the possibility of measuring such a change. Although the long-term impact of smoking and smoking cessation on gingival health and tooth appearance has never been investigated in a prospective trial, the planned 18 months duration of the study is deemed to be adequate for the detection of significant changes in study end points. Chronic periodontal disease is common in smokers and is an irreversible condition that may stabilize with active treatment [52]; however, it is unlikely to improve with the simple measure of smoking cessation alone. Therefore, participants with periodontitis will be excluded and only participants with mild to moderate gingivitis will be recruited, as they are more likely to show measurable changes in study end points with continuing to smoke, smoking cessation, or the use of C-F NA.

Fifth, personal oral hygiene and dietary patterns can significantly influence both primary and secondary study end points. To mitigate this impact, a standardized approach to oral hygiene will be implemented within the study parameters. Participants will be explicitly advised against altering their established oral hygiene practices for the duration of the study. Furthermore, adherence to specific restriction criteria before each scheduled study visit will be emphasized, aiming to prevent any confounding of the collected data. Recognizing the variable effects stemming from individual oral hygiene and dietary habits, we incorporated a cohort of never smokers as a reference group. This inclusion serves to establish a benchmark for comparison and analysis. To address the inherent challenge of ensuring consistent measurement of study end points, especially in cases involving diverse operators across multiple sites, we will provide comprehensive training and implement meticulous calibration procedures.

Finally, compliance with the research protocol is important because it would decrease or nullify the anticipated improvements in study end points if cigarettes were not fully or largely replaced with C-F NA. Participants will be reminded of the importance of adhering to their randomized product allocation and of abstaining from or substantially reducing the daily consumption of cigarettes (by at least 90% of their regular cigarette smoking at baseline) at every study visit. A significant feature of switching studies is close reporting of cigarette consumption and (or) C-F NA use; participants will record the consumption of cigarettes and use of C-F NA on each visit in their study diary. In addition, participants will be asked to return all empty, partly used, and unused consumables. Throughout the study, smoking and C-F NA use will be monitored via an app. The SMILE Tracker app is an integral component of the Smile study designed to monitor participants’ behaviors and lifestyle choices. Through daily prompts, the app assists in tracking cigarette consumption, use of combustion-free nicotine delivery systems, and regular oral hygiene practices such as brushing, flossing, and mouthwash use. In addition, it functions as a personal diary for participants to log any changes in their oral care routine. Moreover, the SMILE Tracker app features a system to monitor protocol adherence, identify deviations, and gather data on potential AEs and SAEs. It sends automated reminders to participants for their upcoming appointments and study restrictions and provides instructions to ensure adherence and accurate data collection during the study. Furthermore, it includes a step counter to encourage physical activity among users. Of note, noncompliance with the study items is an interesting outcome in itself (particularly in consideration of the wide selection of different products offered in the study). Although compliance with this study is not expected to be significantly different compared with other comparable studies, our power calculations are overestimated to account for a 75% noncompliance rate. The C-F NA population would therefore be overrepresented by recruiting4 times the number of participants in the C-F NA category (ie, for every participant randomized in the control population, 4 will be randomized in the C-F NA population).

The acknowledgment of the impact of COVID-19 pandemic restrictions on recruitment is duly noted. However, it is deemed unlikely that these restrictions will exert a significant impact. This assertion is based on the fact that recruitment proceedings were initiated in October 2021, a period during which well-defined guidelines had already been established at the dental clinic across all 4 participating sites.

### Conclusions

This study represents a pioneering effort in assessing the long-term effects of smoking and smoking abstinence on oral health. Through a prospective approach, it aims to provide novel insights into the relationship between smoking habits and oral well-being. The data derived from this study will significantly enhance our existing understanding of the impact of smoking on oral health. Moreover, the study outcomes hold the potential to shed light on the role of C-F NA as a potentially cleaner nicotine alternative to tobacco smoking. This is particularly relevant for individuals who prioritize concerns such as bad breath and dental esthetics. This study’s emphasis on oral health aligns with a broader objective of alleviating the burden of smoking. Several parameters measured in this study (including MGI and dental plaque build-up) are linked to the development of periodontal disease. It is noteworthy that the progression from chronic gingivitis to periodontitis raises the risk of cardiovascular disease [4,5], thereby underscoring the broader health implications of the study’s results.

## Appendix

Multimedia Appendix 1 Study activities—detailed description of the measurements that will be specifically performed at each trial visit.

Multimedia Appendix 2 Study procedures—detailed description of the study measurements that will be specifically carried out at the dental clinic.

Multimedia Appendix 3 CONSORT checklist.

## Abbreviations

AE: adverse event
C-F NA: combustion-free nicotine alternatives
eCRF: electronic case report form
EC: e-cigarette
HTP: heated tobacco product
MGI: Modified Gingival Index
SAE: serious adverse event
VBA: very brief advice
WHO: World Health Organization

## Data Availability

The data sets used during or analyzed during this study will be available from the corresponding author upon reasonable request.
