# Supplementary material for: Changes in Oral Health and Dental Esthetic in Smokers Switching to Combustion-Free Nicotine Alternatives: Protocol for a Multicenter and Prospective Randomized Controlled Trial
Source: JMIR Res Protoc. 2024 Feb 23;13:e53222. doi: 10.2196/53222 (PMC10924261; doi:10.2196/53222)
Supplement: Multimedia Appendix 1 [file resprot_v13i1e53222_app1.docx]

**ANNEX 1** **- Study Activities**

## General Aspects

All participants will have to agree to:

- refrain from scaling and polishing procedures for the whole duration of the study (except for those provided in the protocol)
- avoid modifying their own habitual oral hygiene (e.g., mouthwash, mouth-rinse, interdental floss) for the whole duration of the study.
- refrain from flossing for at least 72 hours prior to each study visit.
- refrain from mouth-rinsing for at least 24 hours prior to each study visit.
- refrain from tooth brushing for at least 2 hours prior to each study visit.
- refrain from eating and drinking (except water) for at least 2 hours before each visit.
- abstain from smoking for 2 hours before each visit.
- complete questionnaire via the automated Tracker App during the entire period of the study.

Participants in Arm A will have to agree to:

- refrain from use of any nicotine or tobacco product other than their own conventional cigarettes brand.

Participants in Arm B will have to agree to:

- refrain from smoking or using any nicotine/tobacco product other than their assigned C-F NA.
- report any change in using their C-F NA or conventional cigarettes (as per automated Tracker App).
- return study devices, chargers and other accessories supplied to them for this study at the last visit.

All visits will need to be conducted in the morning, if possible, around the same time.

## Screening Visit

The participant will be invited to attend a Screening Visit.

The objective of the research will be explained to the participant by a clinical site staff member, and the subject will be asked to take the time to read and review the study-specific Subject Information Sheet.

The subject will be asked to sign the study-specific Informed Consent Form (ICF) in the presence of a clinical site operations staff member prior to any screening procedures being performed. A participant, determined to be eligible during the Screening Visit, is being considered as enrolled into the study after signature of the ICF. Procedures conducted at the Screening Visit are presented in the Schedule of Assessments (see Table 2) and listed here:

- Documentation of socio-demographic data
- Review of smoking history
- Confirmation of participant not intending to quit smoking within the next 30 days - for regular smokers only
- Smoking cessation advice - for regular smokers only
- Number of cigarettes smoked /day
- Carbon Monoxide breath test
- Question of participant about interest in switching to C-F NA (participants will be shown C-F NA devices at screening) - for regular smokers only
- Administration of Fagerström Test for Cigarette Dependence – for regular smoker only
- Documentation of medical history
- Review of Inclusion and Exclusion criteria check list
- Review of pregnancy status by self-reporting
- Review of oral hygiene history by questionnaire (see Appendix 2)
- Periodontal examination
- Instruction of participant to bring the toothpaste they are using to the next visit

## Enrollment and Randomization (Visit 0)

Within 28 days of the screening visit, eligible participants will be invited to attend a study visit. A trained site staff member will perform following tasks (Schedule of Assessments; Table 2):

- Confirmation of participant not intending to quit smoking within the next 30 days - for regular smokers only
- Smoking cessation advice - for regular smokers only
- Number of cigarettes smoked /day
- Carbon Monoxide breath test - for regular smokers only
- Review of Inclusion and Exclusion criteria check list
- Review of pregnancy status by self-reporting
- Review of oral hygiene history
- Participant’s name and brand of toothpaste will be noted
- Randomization of regular smokers to Arm A or B, and assignment of never-smokers to Arm C
- Smokers assigned to Arm B will be asked to familiarize with the C-F NA of their own choice
- Upload of the Tracker APP on participant’s smartphone and complete the questionnaire
- Administration of OHQOL (Oral Health Quality of life) questionnaire
- Administration of EQ VAS – QoL questionnaire
- Measure of vital signs (blood pressure and heart rate)
- Determine height, weight
- Determination of MGI
- Plaque Score Imaging
- Tooth Stains Assessment
- Assessment Dental Discolorations
- Dental Scaling & Polishing to remove plaque, calculus and stains
- Review of AEs and SAEs
- Instruction of participants to bring the toothpaste he/she is using to the next visit

Participants will be reminded not modify their own habitual oral hygiene (e.g., mouthwash, mouth-rinse, interdental floss) pattern for the whole duration of the study. They will be also asked to not change the brand of toothpaste during the study. However, if this has occurred, participants will have to inform the study site investigator.

Smokers randomized into Arm B (switching to C-F NA use) will be asked to try and familiarize themselves with the selection of C-F NA provided by the study investigator in order to choose the product of their preference. They will be trained and counselled on the chosen C-F NA.

After installing the APP for behavioral/lifestyle tracking on participants’ smartphones, they will be given instructions and asked to familiarize themselves with the Tracker APP.

## Baseline Visit (Visit 1)

At day 14, all participants will attend a baseline visit, during which they will undergo the following assessments (Schedule of Assessments; Table 2):

- Smoking cessation advice - for Arm A and B only
- Number of cigarettes smoked /day
- Carbon Monoxide breath test - for Arm A and B only
- Review of oral hygiene history
- Participant’s name and brand of toothpaste will be noted
- Administration of OHQOL (Oral Health Quality of life) questionnaire
- Administration of EQ VAS – QoL questionnaire
- Measure of vital signs (blood pressure and heart rate)
- Determination of MGI
- Plaque Score Imaging
- Tooth Stains Assessment
- Assessment Dental Discolorations
- Switch to C-F NA – for Arm B only
- Check of AEs and SAEs (to be reported in eCRF)
- Instruct participants to bring the toothpaste he/she is using to the next visit

## Visit 2 to Visit 5

Participants will return to the clinic for their scheduled visits and at each visit they will undergo the following assessments (Schedule of Assessments; Table 2):

- Smoking cessation advice - for Arm A and B only
- Number of cigarettes smoked /day
- Review of C-F NA use – for Arm B only
- Carbon Monoxide breath test - for Arm A and B only
- Review of pregnancy status by self-reporting
- Review of oral hygiene history
- Participant’s name and brand of toothpaste will be noted
- Administration of OHQOL (Oral Health Quality of life) questionnaire
- Administration of EQ VAS – QoL questionnaire
- Measure of vital signs (blood pressure and heart rate)
- Determine height, weight
- Determination of MGI
- Plaque Score Imaging
- Tooth Stains Assessment
- Assessment Dental Discolorations
- Check of AEs and SAEs (to be reported in eCRF)
- Instruct participant to bring the toothpaste he/she is using to the next visit (except last visit)

At the end of or after Visit 5, all participants will be offered a final dental scaling & polishing to remove plaque, calculus and stains.
