# Supplementary material for: Changes in Oral Health and Dental Esthetic in Smokers Switching to Combustion-Free Nicotine Alternatives: Protocol for a Multicenter and Prospective Randomized Controlled Trial
Source: JMIR Res Protoc. 2024 Feb 23;13:e53222. doi: 10.2196/53222 (PMC10924261; doi:10.2196/53222)

# Annex 2 - STUDY PROCEDURES

## Specific Restrictions/Requirements

### Oral Hygiene

In relation to their oral hygiene habits (e.g. tooth brushing, mouth rinse, interdental), participants will have to adhere to the following restrictions:

- avoid scaling and polishing procedures for the whole duration of the study (except for those provided in the protocol)
- avoid modifying their own habitual oral hygiene (e.g., mouthwash, mouth rinse, interdental floss) for the whole duration of the study.
- refrain from flossing for at least 72 hours prior to each study visit.
- refrain from mouth rinsing for at least 24 hours prior to each study visit.
- refrain from tooth brushing for at least 2 hours prior to each study visit.

Oral hygiene habits will be documented at the baseline and any change in products has to be reported to the Investigator and documented in the Tracker App.

### Smoking Status

Self-reported smoking status (i.e. number of cigarettes smoked on a daily basis) will be objectively verified by performing an exhaled breath CO test. Regular smokers in Arms A to B will only be enrolled into the study if their exhaled breath CO level is greater than 7 ppm. Participants will be instructed to refrain from smoking for 2 hours prior to each clinical visit.

### Diet and alcohol Restrictions

Participants will be instructed not to eat and drink (except water) for at least 2 hours prior to each day of assessments

## Clinical Assessments

All measurements will be taken at the days indicated in the Schedule of Procedures (Table 1a-c)

### Demographic Data

Socio-demographic data (sex, age, race) will be recorded at the Screening Visit.

### Smoking History and Interest to Switch

Participants will be asked about their smoking history at Screening. This will include questions to:

- evaluate whether the participant has smoked for at least the last 5 consecutive years,
- determine the number of conventional cigarettes smoked by the participant per day on average,
- check that the participant has smoked their chosen brand of conventional cigarette consistently for at least 6 months, and
- determine if the participant regularly uses any nicotine or tobacco product other than their habitual used commercially manufactured filter cigarettes.
- assess whether the participant is planning to quit smoking within the next 30 days
- assess if he/she is interested to switch to C-F NA.

Participant’s name and brand cigarette pack will be noted in the eCRF.

### Medical History and Concomitant Diseases

Co-morbidities and significant, uncontrolled medical condition, as determined by the Investigator, will be documented at screening. Gingivitis, however, will not be added as a co-morbidity in the medical history.

### Vital Signs

Supine blood pressure and supine pulse rate will be measured at the times indicated in the in the schedule of assessments (Table 2) and can also be performed at other times if judged to be clinically necessary.

Blood pressure and pulse rate will be measured using automated monitors. Participants must be at rest and supine for at least 5 minutes before blood pressure and pulse rate measurements.

### Height, Body Weight, and BMI

Height in meters (to the nearest cm), and body weight (BW) in kilograms (to the nearest full kilogram) in underclothing and without shoes will be measured (if possible) or asked to the participant at Screening. The BMI will be calculated by the eCRF by using the formula: BW (kg) / [height (m)]^2^

## Oral Health Parameters and Evaluations

### Modified Gingival Index (MGI)

The MGI, as descripted by Lobene et al. (1986) adopt the following criteria:

- - 0 = absence of inflammation;
  - 1 = mild inflammation or with slight changes in color and texture but not in all portions of gingival marginal or papillary;
  - 2 = mild inflammation, such as the preceding criteria, in all portions of gingival marginal or papillary;
  - 3 = moderate, bright surface inflammation, erythema, edema and/or hypertrophy of gingival marginal or papillary;
  - 4 = severe inflammation: erythema, edema and/or marginal gingival hypertrophy of the unit or spontaneous bleeding, papillary, congestion or ulceration.

The MGI of the individual will be obtained by adding the values of each tooth and dividing by the number of teeth examined. Therefore, the MGI will be assessed in all scorable teeth, except the third molars.

### Dental Plaque Imaging

Fluorescence photographs of the vestibular aspect of the anterior teeth (cuspid to cuspid, upper and lower jaw) in end-to-end position will be taken using Q-ray cam Pro (Co., AIOBIO, Seoul, Republic of Korea) and cheek retractors (Henry Schein, Gillingham, UK, Double end large, 106-7079). The Q-ray cam Pro will be vertically placed to the anterior tooth surface with the cone of the device closely attached to the mouth to take images with a standardized depth of focal length (0.32). Focus will be adjusted to the maxillary lateral incisor and canine.

All the analysis of the QLF-D images will be performed in Catania by a single, blinded operator, using a specific centralized software setting. Dental plaques will be assessed with subscores of ΔR30 and ΔR120, according to the fluorescence intensity. QLF images will not be uploaded in the e-CRF.

### Tooth Stain Assessment

The tooth stain assessment according to Macpherson modification of Lobene [24] consists of dividing each analyzed tooth into 4 separate sites (Fig. 3):

Figure 3.: Macpherson modification of Lobene

1. gingival (G): 2 mm wide strip running parallel to the gingival margin. The limit towards the incisal edge given by the end of the interdental papilla;
2. body of tooth (B): central area of buccal/lingual aspect, between gingival and distal/mesial sites, extending to incisal edge;
3. mesial (M): visible area between line angle and adjacent tooth, ending at the interdental papilla (i.e. start of gingival site);
4. distal (D): as for mesial (M)site

The index consists of a scoring point for each site analyzed representing the intensity and the extent of the stain.

The criteria for the intensity are:

1. no stain present, natural tooth color
2. faint stain
3. clearly visible stain, orange to brown
4. dark stain, deep brown to black

The area criteria and codes extent are:

1. up to 1/3 of area affected
2. between 1/3 and 2/3 of area affected
3. more than 2/3 area affected

Before scoring, participants will be asked to rinse their mouth with water to remove food debris. The index teeth will be dried using a chair-side air syringe and kept dry throughout the examination.

The index will be assessed on the facial surface of the anterior teeth (cuspid to cuspid, upper and lower jaw) recorded and calculating the mean directly on the e-CRF.

### Dental Discolorations

Color change of social six (cuspid to cuspid, up and lower jaw) will be measured using a calibrated spectrophotometer. CIELAB [lightness ranging from 0 (black) to 100 (white)], a* (red/green), b* (yellow/blue)] color parameters will be calculated for each anterior tooth. A standardized protocol for tooth preparation and in vivo spectrophotometric evaluation will be adopted for all participants. Measurements will be performed in the same examination room with standardized lighting conditions by the same operator.

The spectrophotometer will be calibrated according to the manufacturer’s instructions and a sterile mouthpiece will be placed on the optic handpiece for each subject. Three consecutive measurements will be taken for each parameter and the mean after measurements will be considered the baseline value. The CIELab color difference (ΔE*ab) will be calculated as follows: ΔE*ab = [(ΔL*)2 + (Δa*)2 + (Δb*)2]1/2; where ΔL*, Δa*, and Δb* correspond to the difference between each visit and the baseline L*, a*, and b* values, respectively. All the data will be collected as tables of numerical values and directly imported in the e-CRF.

### Periodontal Evaluation

In order to exclude participants with early periodontal disease, participants who meet one of the following clinical periodontal parameters [23] will be excluded from study participation:

- Detectable Interdental Clinical Attachment Loss (CAL) ≥ 3 mm at ≥ 2 non-adjacent teeth.
- Buccal or Oral CAL ≥3 mm with pocketing ≥3 mm detectable at ≥2 teeth.

The observed CAL will be ascribed to non-periodontal causes if it will be aroused by:

1. gingival recession of traumatic origin;
2. dental caries extending in the cervical area of the tooth;
3. the presence of CAL on the distal aspect of a second molar and associated with malposition or extraction of a third molar,
4. an endodontic lesion draining through the marginal periodontium;
5. the occurrence of a vertical root fracture.

The periodontal evaluation will be performed with a standardized periodontal probe in all scorable teeth, except third molars.

Finally, as part of oral hygiene history, the numbers of missing, decayed and filled teeth will be also recorded.

## Other Assessment

### Exhaled Carbon Monoxide

The participants’ smoking status will be objectively verified using an exhaled CO measurement (exhaled CO ≥7 ppm). Exhaled CO (eCO) levels will be measured using a portable CO monitor (MicroCO, Vyaire). Participants will not be allowed to use any study products/smoke any conventional cigarettes within 2 hours prior to CO level measurements.

### Oral Health Quality of Life (OHQoL)

The subjective evaluation of participants’ oral health will be assessed by using the Oral health related quality of life (OHQoL) questionnaire. The questionnaire was developed based on a general population's perceptions of how oral health affects life quality ^(25)^. OHQoL consists of a pool of 3-items: daily work, social activities and communication problems. OHQoL questionnaire will be administered at study visits indicated in the Schedule of Procedures (Table 1 a-c)

### Fagerström Test for Cigarette Dependence (FTCD)

Potential nicotine dependence will be assessed via a questionnaire at Screening and the study visits indicated in the Schedule of Procedures (Table 1 a-c) using a FTCD questionnaire in its revised version (Appendix 4). The questionnaire consists of 6 questions which will be answered by the participant himself/herself. The scores obtained on the test permit the classification of nicotine dependence into 3 levels: Mild (0-3 points), moderate (4-6 points), and severe (7-10 points).

### EuroQol Visual Analog Scale (EQ VAS)

Quality of life and self-reported health condition will be assessed using EQ VAS questionnaire (Appendix 5). EQ VAS questionnaire will be administered at study visits indicated in the Schedule of Procedures (Table 1 a-c)

Figure 3: Macpherson modification of Lobene


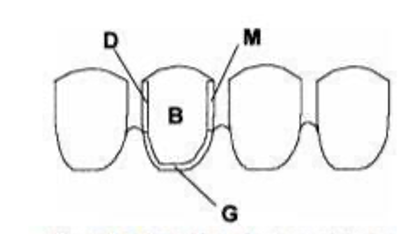

Supplement: Multimedia Appendix 2 [file resprot_v13i1e53222_app2.docx]
